# Young nurses’ perceptions about their employment, working and health conditions *

**DOI:** 10.1590/1518-8345.7398.4335

**Published:** 2024-09-23

**Authors:** Mabel Rocío Hernández Díaz, Zuly Bibiana Suárez Morales, Angelica María Vargas Monroy, Andrey Sebastián Castiblanco Prieto

**Affiliations:** ^1^ Pontificia Universidad Javeriana, Instituto de Salud Pública, Bogotá, DC, Colombia

**Keywords:** Nursing, Working Conditions, Occupational Health, Employment, Health Care Sector, Colombia

## Abstract

**Objective:**

to interpret young nursing professionals’ perceptions about the relationship between working, employment and health conditions.

**Method:**

a qualitative study with an interpretive approach regarding the work-related experiences of 15 young nurses, who took part in the research through voluntary snowball sampling. The data from the interviews and the focus group were analyzed to reach an approximation to the realities inherent to the nurses’ work life.

**Results:**

the relevant study findings pointed out that work precariousness is a characteristic feature in the population group, mainly related to hiring modalities and to wages. According to the participants, the psychosocial working conditions (which were intensified during the COVID-19 pandemic) were the ones that caused the most effects on their mental and physical health.

**Conclusion:**

from the young nurses’ perspective, this study reports how the macro- and micro-structural working conditions and their relationship with health are perceived, pointing out the key elements to devise interventions focused on life paths that foster decent and healthy work postulates in their work environments, as well as actions to prevent injuries or harms to nurses’ health.

## Highlights

**(1)** Young nurses perceive significant work precariousness.

**(2)** The hiring modalities and wages are perceived as the most unfavorable conditions.

**(3)** The participants point out that their employment and working conditions affect their health.

**(4)** According to the participants, the most harmful working conditions are the psychosocial ones.

## Introduction

 The last few decades have witnessed certain deterioration in the Latin American and Caribbean labor market situation, which affects young people. The poor quality of the job positions held by this population group manifests itself in precarious working conditions ^(^ 1 ^)^ , in absence of legal and social protection and in limited opportunities in terms of training and professional development ^(^ 2 ^-^ 3 ^)^ . 

 The conceptual framework of this study is based on the model set forth by Benach ^(^ 4 ^)^ regarding the work-health relationship from the macro- and micro-structural levels. At the macro level, the power relations between governments, companies, union groups and society play a central role in the making of policies that regulate the labor market and the workers’ well-being conditions, thus configuring the employment conditions; in turn, the micro level (that is, working conditions) explains the differential exposure instances of a psychosocial or material nature, as well as their relationship with health. 

 Regarding some working and employment conditions that particularly affect young workers in the health care sector, we can mention the following ones: unstable job positions due to outsourcing and temporary contracts, frequently not duly supervised ^(^ 5 ^)^ ; incorrect decisions on their part for lacking representation and not knowing their labor rights, which can give rise to a tendency to accept unfavorable hiring conditions ^(^ 2 ^)^ ; and lack of job-specific qualification and training opportunities, which indicates that, when incorporating to the workforce for the first time, young people do not have the same competences and experience as adult workers, reason why they are more prone to accepting adverse working and employment conditions ^(^ 3 ^)^ that configure greater work flexibilization and outsourcing of work contracts ^(^ 6 ^-^ 7 ^)^ . 

The aforementioned can become an important precedent that will signal the mediate future, the human talent deficit in the sector and certain impairment in service provision quality, which imposes a harmful potential on health systems.

 Various research studies on precarious working and employment conditions in health professionals point out that they increasingly affect their health, safety and quality of life ^(^ 8 ^-^ 11 ^)^ ; however, they lack the perspective of recently graduated young workers in the human health care area. Therefore, the need emerges to interpret young nursing professionals’ perceptions about the relationship between working, employment and health conditions and, thus, confer visibility to the working conditions that most affect them in order to provide information to the different actors in the health system that allows them to devise actions aimed at improving these conditions. 

## Method

### Type of study

 A qualitative research study with an interpretive approach from Weber’s perspective ^(^ 12 ^)^ , which adopts some phenomenological principles grounded on life experiences from the subjects’ perspective regarding their work contracts, the employment and working conditions they have experienced and how they affect their health. The manuscript followed the recommendations included in the COREQ (Consolidated Criteria For Reporting Qualitative Research) checklist ^(^ 13 ^)^ . 

### **Data collection** *locus* **and period**

The study was carried out from August 2021 to February 2022 in Bogotá, Colombia.

### Participants

In order to seek voluntary participation of both men and women willing to share their experiences, and considering their status as young professionals, an open call was made to nursing professionals through voluntary snowball sampling, by duly announcing the research in unions, nursing schools and graduate networks. The final sample consisted of 15 nurses with no previous link to the researchers and who met the following criteria: being a Nursing professional, being aged between 21 and 28 years old, having worked in Bogotá (Federal District) and having from 4 months to 5 years of work experience.

### Instruments used to collect the data

 Two instruments were employed to collect the data, both subjected to a pilot test. Such test was applied to three interviewees and a focus group in the macro-project of this research, which included five health professionals from areas other than nursing. The data resulting from the pilot test were used to adjust the study from the methodological point of view. Formulation of the questions required in data collection resulted in a review of the pertinent literature and of Benach’s model ^(^ 4 ^)^ , which emerged from an analytical and reflexive action by the researchers. 

Semi-structured interviews: a guide with core questions about their career as care nurses was created, specifically about the job characteristics, as well as of the everyday work activity and the effects on their health, providing the interviewer handling space to moderate the conversation and deepen on the elements that generated greater reactions in the participants, as necessary. The questions that were formulated avoided inducing answers (they were neutral) and the participants were allowed to report their experiences and points of view without issuing any criticism or judgment. Each interview lasted a mean of 120 minutes.

 Focus group: a guide was used ^(^ 14 ^)^ to deepen on and complement certain aspects about the employment, working and health conditions they had to face in their work life, achieving a conversation space among the participants. The focus group lasted 180 minutes. 

### Data collection

As the process to announce the call was developed, a voluntary and for-convenience sample was assembled; this was the first collection moment. A total of 10 individual interviews were conducted, which were scheduled outside the participants’ working hours; this allowed creating a quiet environment with no interruptions that eased dialog and information exchanges in a trustful and empathetic way. From the core elements identified in the interviews, a focus group was carried out with six nurses to deepen on and complement on the information collected. It is worth noting that one of the focus group participants had already been interviewed. The interviews and the focus group were in charge of two researchers, one responsible for their conduction and the other for their recording. In all cases, it was guaranteed that one of the members was trained in Occupational Health and Safety and that the other was duly qualified in Social Sciences, so as to foster integration of perspectives from different disciplines.

 All the professionals had previously signed an informed consent form where they freely and voluntarily expressed their interest in participating in the study. In turn, the research team ensured due anonymity, confidentiality and scientific use of the data collected. The instruments to gather the data were implemented following the Colombian guidelines for data protection, indicating the Personal Data Protection policy of *Universidad Javeriana* in compliance with Law No. 1,581 of 2012 ( *Habeas Data* ) in the informed consent form. 

 Due to the situational context imposed by the COVID-19 health crisis, all the interviews and the focus group were conducted in the remote modality through the *Microsoft Teams* ^®^ tool, which allowed recording them with exclusive access for the lead researcher. The *NVivo* *Transcription* ^®^ plug-in was employed in the *verbatim* transcription of the interviews and the focus group; subsequently, such documents were reviewed by pairs of researchers. 

Remote data collection carefully responded to the pandemic conditions. The authors made reflections about the potential implications of these research studies on the subjects, in relation to issues such as the following ones: 1. guarantees on how their data would be used; 2. application of the results obtained and 3. due respect for the privacy of the experiences shared. Addressing such issues will continue to be a permanent task in qualitative research.

### Data treatment and analysis

 Content analysis ^(^ 15 ^)^ based on reducing and re-elaborating data emerged from the formulation of codes that comprised homogeneous clustering sets. The codes were integrated into categories to establish relationships between them, using axial and selective coding ^(^ 16 ^)^ . 

 The contents from the interviews and the focus group were coded by research team members working in pairs and employing *NVivo 11* ^®^ to reach agreement and therefore validate the result of this activity. Subsequently, code co-occurrence relationships were identified to identify similarities and differences in the perceptions and, thus, establish data saturation. 

**Figure 1 -:**
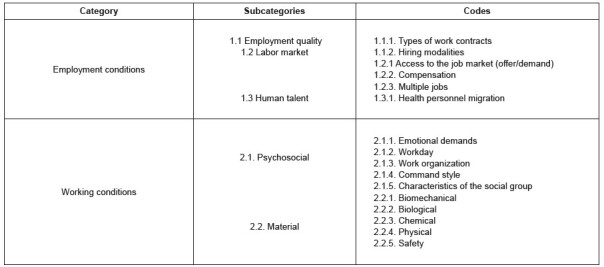
Categories and subcategories identified in the study

Figure 1 presents the categories and subcategories adapted from Benach’s model ^(^ 4 ^)^ to code the data collected. This model defines both the employment conditions and the circumstances in which a person performs a given job or occupation. It frequently supposes that there is some covenant or relationship between an entrepreneur that hires workers and an employee that offers their workforce, as well as the working conditions that include the elements, agents or factors inherent to the work activity and that exert a significant influence on generating risks for workers’ safety and health. 

The emerging category called Pandemic was identified in this study; this category consolidated the characteristics mentioned by the interviews as related to the COVID-19 health crisis.

After coding the information, the data obtained at different moments and spaces with the interdisciplinary research team were interpreted, reaching saturation when information density and authenticity were achieved. The data collected were triangulated, obtaining descriptive information from the participants’ testimonies, which were analyzed in light of the relevant literature about the relationship between employment, working and health conditions. The participants were invited to a socialization event in which the consolidated results from the interviews were disclosed, opening room for discussion in small groups, as well as an exclusive day for nursing professionals through the Colombian Nursing Collegiate Organization.

### Ethical aspects

The research followed the ethical considerations set forth in the Declaration of Helsinki (1964) and in Resolution No. 8,430 (dated 1993) of the Colombian Ministry of Health and Social Protection referring to voluntary participation, confidentiality of the information collected, application of informed consent forms and storage of the data obtained.

The field work was developed considering the safety, voluntary consent and autonomy principles. The data collected was handled in a confidential and anonymous way and solely for the research purposes, deleting all files once they were transcribed.

 The research project was approved by the Research and Ethics Committee of the Public Health Institute belonging to *Pontificia Universidad Javeriana* – Bogotá, Colombia. 

## Results

### Sociodemographic and occupational characteristics

**Figure 2 -:**
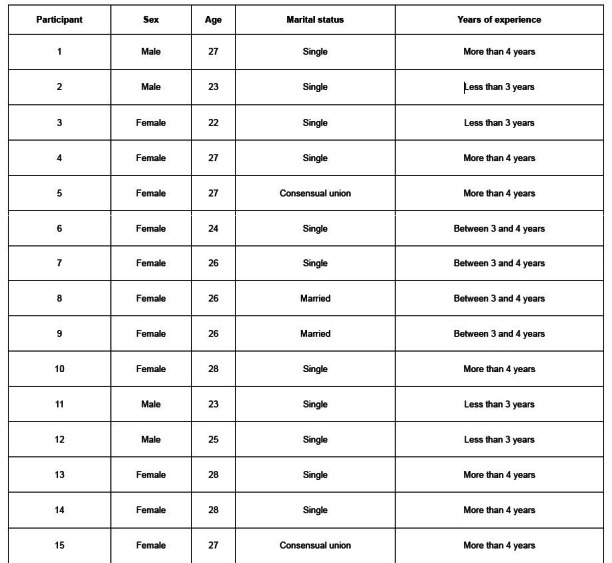
Sociodemographic characteristics of the participating young nurses (n = 15). Bogotá, Colombia, 2021-2022

Figure 2 presents the characteristics of the research with the participation of 15 nursing professionals: 73% of them self-identified as belonging to the female gender and were single; their mean age was 25.8 years old (±2); and 47% had more than four years of work experience. 

 From the occupational point of view, it was found that the participants’ mean monthly income varied between USD 452 and USD 904 ^1^ , their workday presented a daily range from 65 to 12 hours and the most frequent work areas were Hospitalization, Urgencies, Intensive Care Unit and External Consultations. 

### Employment conditions

**Figure 3 -:**
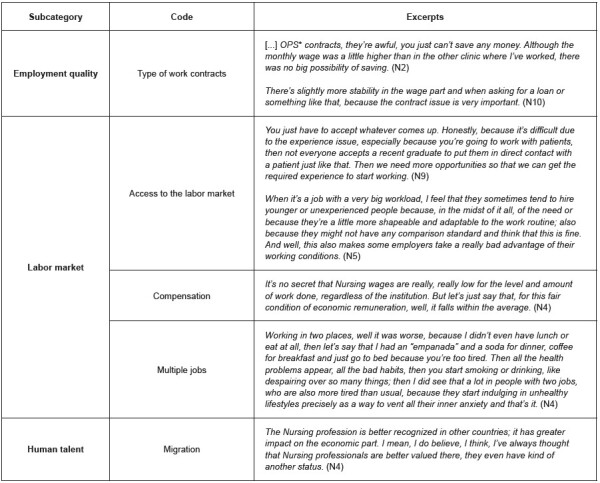
Excerpts from the interviews referring to the young nurses’ perceptions about the employment conditions (n = 15). Bogotá, Colombia, 2021-2022

 Regarding the employment quality subcategory described in Figure 1 , most of the interviewees commented that more than 80% of the young professionals in their workplaces are hired through service provision contracts ^2^ , although none of them has this this type of contract (see the related referents in Figure 3 ). They also indicate absence of social benefits such as paid holidays, primes and sacking, among others, that limit their future projects and saving intentions. 

The overall perception that these are the most common types of contracts and those with the most disadvantages was found in the focus group. Work instability, no paid holidays and impossibility of saving and obtaining loans are among the disadvantages.

 According to Figure 1 , access to the labor market and compensation were the most cited among the codes related to the labor market subcategory. For the interviewees, their young age determines their possibility to access a decent job. They perceive that well-paid positions require an experience level that they simply cannot have acquired due to their age. This closes doors on them and even vacancies for which they consider to be duly qualified, leading them to accept precarious job offers. 

In general, the professionals interviewed consider that the economic compensation they earn for their work is low in relation to the number of activities they perform and the responsibility inherent to them. Even those that feel satisfied with their income usually state that, when they compare it with their acquaintances’ wages, health professionals are ill-paid.

 Multiple jobs were the situation most discussed among the participants, which indicates that it is not an uncommon activity for health professionals. Almost all the individuals interviewed had undergone the experience or knew someone who had more than one job. In fact, one of the interviewees stated that this activity is colloquially known as *turnear* ( *taking turns* ). 

The professionals that accept two jobs do so because the wages are usually low and due to work instability. In addition, they find it feasible because health institutions frequently organize work into morning and night shifts, which allows many professionals to work in a given place during the morning and in another one at the end of the day.

*OPS = *Orden de Prestación de Servicios* (Service Provision Order)

 Several participants expressed their wish to migrate; this code corresponds to the human talent subcategory presented in Figure 1 . The main reason is perceiving that, in addition to better living conditions than in Colombia, richer countries such as Germany, United States of America or Canada offer better working conditions and greater social recognition for their professions. Not speaking foreign languages, being rooted to the country and family, migration uncertainty and underemployment are some of the factors that limit migration. 

For Nursing professionals, the physical discomforts related to their job can be associated with negative effects on their life habits and everyday care practices. Stress appears as a factor that triggers headaches or stomachaches, which are associated with the workloads faced by these professionals.

The distress related to insufficient money to meet their economic obligations and the frustration associated with lack of independence were mentioned as some of the most frequent symptoms that affected their mental health.

Likewise, the interviewees consider that having multiple jobs affects both the professionals and the quality of the services provided, as the former sleep less and are more tired and less willing to work.

In general, other physical discomforts such as tiredness, muscle pain and migraines are found in professionals with at least two jobs because they need to report to two bosses, as well as due to increased commute times, administrative and mental loads, and insufficient time for daily rest, leisure and healthy habits.

### Psychosocial working conditions

**Figure 4 -:**
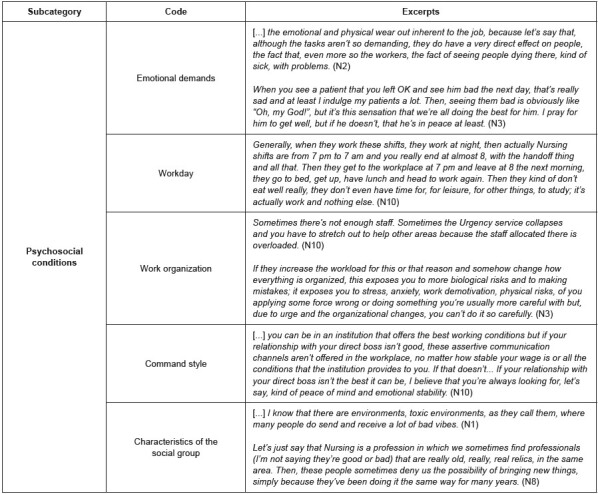
Excerpts from the interviews referring to the young nurses’ perceptions about the psychosocial working conditions (n = 15). Bogotá, Colombia, 2021-2022

Figure 4 shows the excerpts referring to the psychosocial conditions identified by the participants, included in the working conditions category described in Figure 1 . They were varied and alluded to various issues that represented discomfort or concern sources. First and foremost, the professionals pointed out the mental demands of attention, concentration and memory involved in the clinical and administrative activities inherent to caring for the health of other people as a relevant psychosocial condition. Likewise, they identified high emotional demands as a result of their exposure to diseases and death, as well as for witnessing the patients’ distress every day, which is intensified by the emotional connection or bond they establish with them. The participants associated high mental load and emotional demands to effects such as fatigue, tiredness, frustration and somatization, which was especially exacerbated during the pandemic. 

On the other hand, shift turnover was associated with a greater adaptive effort with negative implications on the professionals’ habits and lifestyles, as well as in their interpersonal relationships outside the workplace. Shift variability was identified as an aspect that hinders establishing routines, maintaining good habits and developing activities other than work.

 In addition, it was mentioned that the administrative decisions and changes in work organization have negative repercussions for them to develop their activities, which has oftentimes imposed certain overload or unequal work distribution; this was associated with wear out, fatigue and tiredness, translated by the participants as work-related *burnout* . 

According to the professionals, the interpersonal relationships with their peers in the institution where they work define how they develop the activities, their job satisfaction and even their mood. This is because conflicts between peers and deficiencies in communication create a tense and stressful environment. The aforementioned can be intensified due to factors such as seniority and differences in terms of years of work experience, where it is more difficult to relate to more experienced colleagues.

Finally, the relationship with direct bosses was identified as an aspect carrying plenty of weight in overall satisfaction and well-being in the workplace. Therefore, command styles characterized by good communication and support were perceived as extremely favorable; on the other hand, deficiencies in communication and lack of assistance and support are perceived as characteristics of leadership styles that exert a negative impact among the professionals.

### Material working conditions

**Figure 5 -:**
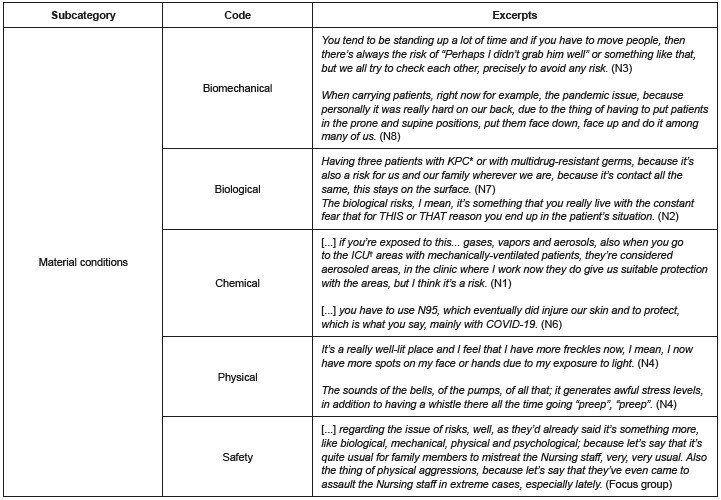
Excerpts from the interviews and focus group referring to the young nurses’ perceptions about the material working conditions (n = 15). Bogotá, Colombia, 2021-2022

Figure 5 presents excerpts from the participants’ testimonies corresponding to the material working conditions (see Figure 1 ), where the most relevant ones will be mentioned. In relation to exposure to biomechanical danger, the professionals stated that they frequently perform their tasks in a standing position, followed by sitting on inadequate work surfaces, reason why they have to adopt unnatural and sustained positions during their workday, with the consequent discomforts or pain. 

Physical effort and load handling were mentioned in the task of moving patients in emergency situations or those with mobility restrictions. In addition, repeated movements were perceived in two situations, namely: opening vials while administering medications; and using a computer for the administrative activities.

According to the characterization of biological danger, the nurses mentioned certain normalization of such exposure due to their professional duties; special allusion was made to handling patients with confirmed multidrug-resistant micro-organisms as risk situations. They also mentioned moments marked by uncertainty, anguish and fear regarding the possibility of contracting infections when exposed to accidents while handling contaminated objects.

*KPC = *Klebsiella pneumoniae carbapenemase* ; ^†^ ICU = Intensive Care Unit

The participants stated that several occupational risks were intensified during the pandemic, namely: extremely high exposure to SARS-CoV-2; thermal discomforts due to overcrowding of patients; deficient ventilation in care provision areas; increased hand hygiene frequency; handling of patients because of their critical condition that prevents them from moving; and permanent use of personal protective equipment, which imposed changes in their everyday habits.

Other situations imposing risks to the participants’ health consisted in chemical products as a result of hand hygiene, use of latex gloves and handling of medications, exposure to noise or to inadequate lighting, handling of beds or wheelchairs in poor condition, the assistance provided to patients and interactions with family members due to possible aggressions (blows and bites).

## Discussion

 The sociodemographic characterization identified in the study corroborates the predominance of the female sex in the nursing profession. The relevant findings of the study related to the young nurses’ perceptions about their employment, working and health conditions indicated that work precariousness is a characteristic feature of the health care sector due to the low probability of finding a job with occupational guarantees because of the types of contracts and the wages offered in the labor market ^( 5^ , ^9^ )^, which fosters multiple jobs among nurses ^(^ 17 ^)^ . 

 Based on the model set forth by Benach ^(^ 4 ^)^ , which allows us to interpret the young nursing professionals’ perceptions about the relationship between working, employment and health conditions from the macro- and micro-structural levels, the research findings allowed identifying how work precariousness in the health care sector reflects the labor market conditions. Two aspects that contribute to work precariousness draw the attention, namely: outsourcing versus direct hiring by the health institutions, which has increased unaccountability on their part to ensure dignified employment conditions; and exploitation by the employers by hiring young professionals with a need to accrue work experience, conditions that are analyzed in other studies, where age and being a recent graduate are relevant in the hiring of services in the health care sector ^(^ 3 ^)^ . The aforementioned exerted some effects on the participants’ health, as they mentioned psychological discomforts such as stress and anxiety with somatic manifestations like headaches/pain in other body parts and insomnia. 

 On the other hand, both wages lower than their expectations as nursing professionals in line with the workload and the schooling level attained ^(^ 18 ^)^ and unawareness about the labor market conditions in recent graduates were situations that generated various psychological discomforts in them, such as anxiety or demotivation. These aspects lead them to think about migrating with the purpose of improving their quality of life. 

 From the perspective of the working conditions set forth by Benach ^(^ 4 ^)^ in the micro-structural component of his model, the nurses’ psychosocial conditions are characterized by the requirement of developing work activities in rotating and night shifts, having to work on days usually devoted to rest, working long and extenuating workdays, and experiencing frequent timetable changes (work shift extension or changes in the workday start and end times), which imposes difficulties for them to develop their personal and out-of-the-job activities and impairs the balance between family, social and work life ^(^ 3 ^,^ 19 ^-^ 21 ^)^ . 

 The direct assistance provided to patients and the frequent interactions with family members are a distinctive and inherent characteristic of the care practice in this profession, which involves a series of emotional demands inherent to working with people in a situation of vulnerability. Among the specific elements of the emotional demands we can mention the following ones: close coping with diseases and death; handling of patients and family members with altered emotions (nervous, fearful, anxious or stressed), reason why they find it overwhelming to interact with them; the permanent requirement of understanding and empathizing with the fear, anxiety and distress of patients and relatives alike; and the need to control and hide their own emotions of fear, uncertainty, pain and frustration that can emerge while performing their duties ^(^ 22 ^-^ 23 ^)^ . It is worth noting that direct interpersonal contact with patients and family members is conducive to a context in which instances of violence are materialized in a really varied way, such as verbal abuse, intimidation, threats, sexual harassment and physical aggressions ^(^ 24 ^-^ 25 ^)^ . 

 The mental load demand, associated with the high requirements in terms of concentration, attention and memory imposed on nurses due to their job, as well as the demands derived from handling detailed and precise information, is another distinctive psychosocial risk factor. It is warned that, when this condition presents itself constantly in the workplace, such circumstance can be associated with deficits in work performance, fatigue or tiredness and higher chances of making mistakes ^(^ 24 ^)^ . 

 The relationships or contact that are established with other people in the work environment represent multiple types of interactions for nurses, such as those with bosses or superiors, peers working in care or administrative areas, patients and their relatives and external people. All of them can mean a source of danger because they are usually developed under time constraints, high emotional loads, high work volumes and atypical work schedules. This context is described as a conducive setting for deficient social support from bosses and among peers, ineffective communication and difficulties and conflicts in interpersonal relationships with bosses, peers or professionals from other work areas ^(^ 22 ^-^ 23 ^,^ 25 ^-^ 26 ^)^ . 

 In all, the material working conditions in the Nursing profession represent a high risk of developing musculoskeletal disorders (back, neck, shoulder and lower limb pain) due to the need to grab and move patients with restricted mobility, to remain in a seated position, walk and standing up for extended periods of time, and to push or pick up heavy objects and equipment, among other situations ^(^ 27 ^-^ 29 ^)^ . 

 In line with other studies, inadequate workplaces or ill-designed furniture (stretchers and chairs) were one of the reasons for the ailments, identifying a relationship between musculoskeletal injuries and working conditions ^(^ 29 ^)^ . The presence of musculoskeletal symptoms was underestimated by the young professionals, who did not judge them as sufficiently severe or painful to report them to their employers, attributing them to incorrect positions and to risky work practices ^(^ 30 ^)^ . 

 Another subtopic of interest was exposure to infectious agents due to contact with blood and bodily fluids, although the interviewees stated naturalizing permanent and direct contact with these micro-organisms in their routine activities ^(^ 31 ^)^ . A phenomenological study conducted with ten female nurses in Iran showed the high risk of exposure to blood-borne and hospital-acquired diseases ^(^ 28 ^)^ . Biological accidents due to punctures with needles and sharps are usual among nurses ^(^ 32 ^-^ 33 ^)^ ; in the current study, all the participants stated having had at least one accident during their career ^(^ 33 ^)^ . 

 Although present, the participants did not identify perceptions about other working conditions such as temperature, lighting, noise and drug handling so frequently ^(^ 34 ^-^ 35 ^)^ . 

 The COVID-19 pandemic imposed changes both in the employment and working conditions in the Nursing profession. Despite a remarkable increase in the job offer to respond to the mass care demand, the contracts only lasted a few months, which generated economic insecurity in the long term ^(^ 3 ^)^ . In addition, although their wages and employment quality remained low, the nurses mentioned that the workload was increased during this period ^(^ 17 ^)^ . 

 Multiple studies have shown exacerbation of physical and psychological discomforts related to increased workloads and extended schedules ^(^ 36 ^-^ 37 ^)^ , which resulted in changes in life and work styles ^(^ 38 ^-^ 39 ^)^ . During the pandemic, dermatitis or skin lesions due to intensive hand washing (over ten times a day) and mask use for more than six hours were relevant ^(^ 32 ^,^ 39 ^)^ and were associated with the fear of contracting infections and transmitting them to friends and family members ^(^ 35 ^)^ . 

Regarding the contributions of this study to scientific advance for the nursing profession, we should first acknowledge the value of qualitative research to understanding the work-health relationship beyond risk quantification, rescuing the young nurses’ voice and their perceptions, where the types of contract and the wages predominantly stand out, as well as the psychosocial factors, which are the ones most affecting their health. Likewise, the study findings contribute an integrated analysis between the employment and working conditions, overcoming the traditional view of occupational safety and health, in addition to incorporating the factors that reflect the power relations between the government, the employers and the professionals.

Based on the findings presented in this research, the need emerges to consider in nursing professionals’ training processes the possibility of including spaces targeted at understanding the factors that determine recent graduates’ inclusion in the labor market, in order to prepare them to face it. Likewise, there is an evident need to train them to carry out prevention actions against exposure to psychosocial, biomechanical, biological and safety conditions in their workplaces, as they are priorities in the health care sector.

It is worth noting that the analysis by pairs and the fact of having assembled an interdisciplinary team with limited experience in the care field allowed placing the participants’ discourse in the forefront and eased the reflections about their working conditions and being young in the labor market from various perspectives.

The study methodological scope and planning imposed some limitations that may be overcome in subsequent surveys, namely: a) the sample lacked greater diversity in relation to the hiring modalities to enrich the findings; b) non-representativeness of nurses from other Colombian regions, as the employment and working conditions can vary substantially; c) cross-sectionality of personal reflexivity in the research team to state the influence in data collection and analysis beyond the professional perspective; d) remote conduction of the focus group and e) the fact that the participants’ findings were acknowledged individually.

## Conclusion

Most of the participants considered that their job has exerted a negative effect on their mental, emotional and physical health and also on their healthy life habits, which generates stress or anxiety, feelings of uncertainty and work insecurity due to hiring instability.

Young workers usually accept job opportunities due to their need to have certified work experience. The importance of work stability is mentioned when it comes to choosing a job since, in addition to providing peace of mind, it enables their independence faster.

As for the working conditions, the psychosocial ones were the most relevant for the professionals, as the workdays, rotating and night shifts, interactions with other people and mental load demands alter the way in which work, personal and out-of-the-job activities are developed, which were exacerbated during the COVID-19 pandemic.

## Footnotes

1 American dollar quote = COP$ 3,981.16 as of 12/31/2021.

2 In Colombia, hiring by service provision corresponds to the type of contract where there is no subordination, reason why each worker enjoys independence and autonomy. People earn their wages as fees, and each interested person has to quote this amount and pay their social security and benefits (Article 34 from the Labor Substantive Code).
