# Supplementary material for: Young nurses’ perceptions about their employment, working and health conditions
Source: Rev Lat Am Enfermagem. 2024 Sep 23;32:e4335. doi: 10.1590/1518-8345.7398.4335 (PMC11421518; doi:10.1590/1518-8345.7398.4335)
Supplement: Translation

# Translation: Percepciones de jóvenes enfermeros sobre sus condiciones de empleo, trabajo y salud *

**DOI:** 10.1590/1518-8345.7398.4334

**Published:** 2024-09-23

**Authors:** Mabel Rocío Hernández Díaz, Zuly Bibiana Suárez Morales, Angelica María Vargas Monroy, Andrey Sebastián Castiblanco Prieto

**Affiliations:** ^1^ Pontificia Universidad Javeriana, Instituto de Salud Pública, Bogotá, DC, Colombia

**Keywords:** Enfermería, Condiciones de Trabajo, Salud Ocupacional, Empleo, Sector Salud, Colombia

## Abstract

**Objetivo:**

interpretar las percepciones que tienen los profesionales jóvenes de enfermería de la relación entre condiciones de trabajo, empleo y salud.

**Método:**

estudio cualitativo con abordaje interpretativo de las experiencias laborales de 15 enfermeros jóvenes, quienes participaron de la investigación a través de un muestreo voluntario por bola de nieve. Se realizó análisis de datos de las entrevistas y grupo focal para lograr una aproximación a las realidades de la vida laboral.

**Resultados:**

los hallazgos relevantes del estudio señalaron que la precarización laboral es característica en este grupo poblacional, relacionada principalmente con tipo de contratación y retribución recibida. Las condiciones de trabajo psicosociales fueron las que más generaron efectos en la salud mental y física según los participantes, las cuales se agudizaron durante la pandemia por COVID-19.

**Conclusión:**

este estudio aporta, desde la perspectiva de los jóvenes enfermeros, cómo se perciben las características macro y microestructurales del trabajo y su relación con la salud, señalando los elementos clave para generar intervenciones con enfoque de curso de vida que promulguen los postulados de trabajo decente y saludable en sus entornos de trabajo, al igual que acciones para la prevención de lesiones o daños en la salud de los enfermeros.

## Puntos destacados

**(1)** Los jóvenes enfermeros perciben una precarización laboral significativa.

**(2)** Los tipos de contratación y la remuneración se perciben como las condiciones más desfavorables.

**(3)** Los participantes indican que las condiciones de empleo y de trabajo afectan su salud.

**(4)** Las condiciones de trabajo psicosociales son las más nocivas según los participantes.

## Introducción

 En las últimas décadas se ha presentado un empeoramiento de la situación del mercado laboral en América Latina y el Caribe que afecta a los jóvenes. La mala calidad de los empleos de este grupo poblacional se manifiesta en las condiciones de trabajo precarias ^(^ 1 ^)^ , la falta de protección jurídica y social, y las limitadas oportunidades de formación y de desarrollo profesional ^(^ 2 ^-^ 3 ^)^ . 

 El referente conceptual de este estudio está basado en el modelo planteado por Benach ^(^ 4 ^)^ de la relación trabajo-salud desde los niveles macro y microestructural. En el nivel macro las relaciones de poder entre los gobiernos, las empresas, los grupos representantes de los trabajadores y la sociedad juegan un rol central en la generación de políticas que regulan el mercado laboral y las condiciones de bienestar de los trabajadores, configurando las condiciones de empleo; mientras que el nivel micro, esto es, condiciones de trabajo, explica las exposiciones diferenciales de carácter psicosocial o material, y su relación con la salud. 

 Con referencia a algunas condiciones de trabajo y empleo que afectan de forma particular a los trabajadores jóvenes del sector salud, se encuentran: los empleos inestables por la tercerización y temporalidad siendo frecuente que estos no estén supervisados apropiadamente ^(^ 5 ^)^ ; decisiones equivocadas de ellos por carecer de representación y desconocer sus derechos laborales, lo que puede provocar la tendencia a aceptar condiciones desfavorables de vinculación laboral ^(^ 2 ^)^ ; y la falta de oportunidades de cualificación y formación para el trabajo, lo que indica que una persona joven al incorporarse por primera vez a la fuerza de trabajo, no tiene las mismas competencias y experiencia que un trabajador adulto, debido a lo cual es más proclive a aceptar condiciones de trabajo y empleo adversas ^(^ 3 ^)^ que configuran una mayor flexibilización laboral y tercerización de la relación laboral ^(^ 6 ^-^ 7 ^)^ . 

Lo anterior puede constituirse en un precedente importante que marcará en el futuro mediato, el déficit del talento humano en el sector y el detrimento de la calidad de la prestación de servicios, lo que implica un potencial nocivo sobre los sistemas de salud.

 Diversas investigaciones realizadas sobre las condiciones de trabajo y empleo precarias en profesionales de la salud señalan que cada vez más su salud, seguridad y calidad de vida se afectan ^(^ 8 ^-^ 11 ^)^ , sin embargo, estas adolecen de una perspectiva desde los trabajadores jóvenes del área de cuidado de la salud humana que han egresado recientemente. Surge entonces la necesidad de interpretar las percepciones que tienen los profesionales jóvenes de enfermería de la relación entre condiciones de trabajo, empleo y salud, y de esta manera visibilizar las condiciones laborales que más los afectan, para brindar información a los diferentes actores del sistema de salud que les permita generar acciones que tiendan a mejorar estas condiciones. 

## Método

### Tipo de estudio

 Investigación cualitativa, con abordaje interpretativo desde la perspectiva de Weber ^(^ 12 ^)^ , que acoge algunos principios fenomenológicos fundamentándose en las experiencias de vida desde la perspectiva del sujeto, en torno a su vinculación laboral, las condiciones de empleo y de trabajo que han vivenciado y cómo estas afectan la salud. El manuscrito siguió las recomendaciones de la lista de comprobación de criterios COREQ ( *Consolidated Criteria For Reporting Qualitative Research* ) ^(^ 13 ^)^ . 

### Lugar y periodo de recolección de los datos

El estudio se realizó en Bogotá, Colombia, desde agosto de 2021 a febrero de 2022.

### Participantes

Con el fin de buscar la participación voluntaria de hombres y mujeres, que tuvieran disposición de compartir sus experiencias, teniendo en cuenta la condición de ser profesional joven, se realizó una convocatoria abierta a profesionales de enfermería a través de un muestreo voluntario y por bola de nieve, mediante la divulgación en agremiaciones, facultades de enfermería y redes de egresados. Se logró contar con la participación de 15 enfermeros, quienes no tenían relación previa con los investigadores y cumplían los siguientes criterios: ser profesional titulado en enfermería, tener entre 21 y 28 años de edad, haber trabajado en Bogotá, D.C., con mínimo de 4 meses y máximo de 5 años de experiencia laboral.

### Instrumentos para recopilar datos

 Se emplearon dos instrumentos para la recolección de datos sometidos a una prueba piloto. Dicha prueba se aplicó a tres entrevistados y un grupo focal en el proyecto marco de esta investigación, que contempló cinco profesiones de la salud diferentes a enfermería. Los datos resultados del piloto fueron usados para el ajuste metodológico del estudio. La construcción de las preguntas requeridas en la recolección de datos resultó de la revisión de la literatura y del modelo Benach ^(^ 4 ^)^ , las cuales emergieron de un ejercicio analítico y reflexivo de los investigadores. 

Entrevistas semi-estructuradas: se diseñó una guía de preguntas centrales sobre su trayectoria laboral como enfermeros asistenciales, específicamente, sobre las características del empleo, de la actividad laboral cotidiana y los efectos en su salud, dando espacio de manejo al entrevistador para orientar la conversación y profundizar los elementos que generaron mayores reacciones en los participantes, según fuera necesario. Las preguntas formuladas evitaron inducir respuestas, siendo neutrales y se permitió a los participantes narrar sus experiencias y puntos de vista sin emitir críticas o juicios al respecto. Cada entrevista duró en promedio 120 minutos;

 Grupo focal: se contó con una guía orientadora ^(^ 14 ^)^ para profundizar y complementar aspectos sobre las condiciones de empleo, trabajo y salud que enfrentaron en su vida laboral, logrando un espacio de conversación entre los participantes. La duración del grupo focal fue de 180 minutos. 

### Recolección de datos

A medida que se surtía el proceso de divulgación de la convocatoria, se fue conformando la muestra voluntaria por conveniencia, siendo éste el primer momento de recolección. Se realizaron en total 10 entrevistas individuales, las cuales se programaron fuera de la jornada laboral, lo cual permitió crear un ambiente tranquilo sin interrupciones que facilitó el diálogo e intercambio de información con confianza y empatía. A partir de los elementos centrales identificados en las entrevistas, se llevó a cabo un grupo focal con la participación de seis enfermeros, para profundizar y complementar la información recopilada. Cabe anotar que uno de los participantes del grupo focal ya había sido entrevistado previamente. La dirección de las entrevistas y el grupo focal contó con dos investigadores, uno responsable por su conducción y el otro por su registro. En todos los casos se garantizó que uno de los integrantes tuviera formación en seguridad y salud en el trabajo y el otro, formación en ciencias sociales para fomentar la integración de diferentes perspectivas disciplinares.

 Todos los profesionales firmaron previamente un consentimiento informado, en el que expresaron de forma libre y voluntaria su interés en participar en el estudio. Por su parte, el equipo investigador garantizó las condiciones de anonimato, confidencialidad y uso científico de los datos recolectados. Los instrumentos para la recolección de datos se implementaron siguiendo las directrices colombianas de protección de datos, indicando en el consentimiento informado la política de la Universidad Javeriana de Protección de datos personales en cumplimiento de la Ley 1581 del 2012 ( *Habeas Data* ). 

 Debido al contexto situacional de la crisis sanitaria por COVID-19, todas las entrevistas y el grupo focal fueron realizados de forma remota utilizando la herramienta *Microsoft Teams* ^®^ , lo que permitió su grabación con acceso únicamente al investigador principal. La transcripción textual de las entrevistas y del grupo focal empleó el complemento *NVivo* *Transcription* ^®^ , posteriormente se realizó una revisión de dichos documentos por duplas de investigadores. 

La recolección de datos de manera remota respondió cuidadosamente a las condiciones de pandemia. Los autores formularon reflexiones sobre las implicaciones que estas investigaciones podrían tener en los sujetos en relación con asuntos como: 1. La garantía en el uso de sus datos; 2. La aplicación de los resultados obtenidos; 3. El respeto por la privacidad de sus experiencias compartidas. Abordar estas cuestiones seguirá siendo una tarea permanente en la investigación cualitativa.

### Tratamiento y análisis de datos

 El análisis de contenido ^(^ 15 ^)^ basado en la reducción y reelaboración de datos surgió a partir de la formulación de códigos que conformaron conjuntos homogéneos de agrupamiento. Se integraron los códigos en categorías para establecer relaciones entre ellos, utilizando codificación axial y selectiva ^(^ 16 ^)^ . 

 La codificación de los contenidos de las entrevistas y grupo focal fue realizada por los integrantes del equipo investigador en duplas, empleando *NVivo 11* ^®^ con el fin de lograr concordancia y validar así el resultado de esta actividad. Seguidamente, se identificaron relaciones por cocurrencia de los códigos para identificar similitudes y diferencias en las percepciones y establecer así saturación de los datos. 

 En la Figura 1 se presentan las categorías y subcategorías adaptadas del modelo de Benach ^(^ 4 ^)^ para la codificación de los datos recopilados. En este modelo se definen las condiciones de empleo como también las circunstancias en las que una persona realiza un trabajo u ocupación. Supone con frecuencia la existencia de un acuerdo o relación entre un empresario que contrata a los trabajadores y un empleado que ofrece su fuerza de trabajo y las condiciones de trabajo que contemplan aquellos elementos, agentes o factores propios de la actividad laboral que tienen influencia significativa en la generación de riesgos para la seguridad y salud de los trabajadores. 

**Figura 1:** - Categorías y subcategorías del estudio

Se identificó en este estudio la categoría emergente denominada pandemia, en la que se consolidaron las características que refieren los entrevistados relacionadas con la crisis sanitaria por COVID-19.

Después de la codificación de la información, se interpretaron los datos obtenidos en diferentes momentos y espacios con el equipo interdisciplinario de la investigación, alcanzándose la saturación cuando la densidad y autenticidad de la información se logró. Se triangularon los datos recolectados, obteniendo información descriptiva desde las narrativas de los participantes, que se analizaron a la luz de la literatura relevante alrededor de la relación entre las condiciones de empleo, trabajo y salud. Se convocó a los participantes a un evento de socialización en el que se dieron a conocer los resultados consolidados de las entrevistas abriendo un espacio para discusión por pequeños grupos, y una jornada exclusiva para los profesionales de enfermería a través de la Organización Colegial de Enfermería en Colombia.

### Aspectos éticos

La investigación se enmarcó bajo las consideraciones éticas establecidas por la Declaración de Helsinki (1964), y la Resolución 8430 de 1993 del Ministerio de Salud y Protección Social de Colombia, en lo referente a la participación voluntaria, la confidencialidad de los datos recopilados, la aplicación de consentimiento informado y el almacenamiento de la información obtenida.

El trabajo de campo se desarrolló considerando los principios de seguridad, consentimiento voluntario y autonomía. Los datos recolectados fueran manejados de forma confidencial, anónima y sólo para efectos de la investigación, eliminando los archivos una vez transcritos.

El proyecto de investigación fue aprobado por el Comité de Investigación y Ética del Instituto de Salud Pública de la Pontificia Universidad Javeriana – Bogotá, Colombia.

## Resultados

### Características sociodemográficas y ocupacionales

 La Figura 2 presenta las características de la investigación donde participaron 15 profesionales de enfermería, 73% de ellos se identificaron con el sexo femenino y estaban solteros, la media de edad fue de 25,8 años (± 2) y el 47% contaba con experiencia laboral mayor a cuatro años. 

**Figura 2:** - Características sociodemográficas de los enfermeros jóvenes participantes (n = 15). Bogotá, Colombia, 2021-2022

 Ocupacionalmente, se encontró que los ingresos mensuales oscilaban entre USD 452 a 904 ^1^ , la jornada de trabajo tuvo un rango diario de 6 a 12 horas y las áreas de trabajo más frecuentes fueron hospitalización, urgencias, unidad de cuidados intensivos y consulta externa. 

### Condiciones de empleo

 Con respecto a la subcategoría de calidad de empleo descrita en la Figura 1 , la mayoría de los entrevistados comentaron que en sus lugares de trabajo más del 80% de los profesionales jóvenes son vinculados a través de contratos de prestación de servicios ^2^ , a pesar de que ninguno de ellos tiene este tipo de contrato (ver los referentes relacionados en la Figura 3 ). Manifiestan también la ausencia de prestaciones sociales como vacaciones remuneradas, primas, cesantías y otros beneficios que limitan sus proyectos a futuro y la intención de ahorro. 

En el grupo focal se encontró la percepción generalizada de que estos son los tipos de contratos con más desventajas y más comunes. Dentro de las desventajas, se menciona la inestabilidad laboral, la falta de vacaciones pagas, la imposibilidad de ahorrar y obtener créditos.

 Dentro de los códigos relacionados con la subcategoría de mercado laboral de acuerdo con la Figura 1 , el acceso al empleo y la retribución fueron los más referidos. Para los entrevistados, ser jóvenes determina sus posibilidades de acceso a un empleo justo. Ellos perciben que los empleos bien remunerados requieren experiencia que, por su edad, les es imposible haber conseguido. Esto les cierra las puertas, incluso, de vacantes para las cuales se consideran capacitados llevándolos a aceptar ofertas de trabajo precarias. 

En general, los profesionales entrevistados consideran que la remuneración económica que reciben por su trabajo es baja para la cantidad de actividades que realizan y la responsabilidad de las mismas. Incluso quienes se sienten satisfechos con sus ingresos, suelen decir que, al compararse con lo que ganan sus conocidos, las profesiones de la salud están mal remuneradas.

 El pluriempleo fue la situación más conversada entre los participantes, lo que indica que esta es una actividad común dentro de los profesionales del sector salud. Casi que todas las personas entrevistadas habían tenido la experiencia o tenían alguien cercano que lo hacía. De hecho, uno de los entrevistados manifestó que a esta actividad se le conoce coloquialmente como *turnear* . 

Los profesionales que asumen dos trabajos lo hacen porque la remuneración de los empleos suele ser baja y existe inestabilidad laboral. Además, es posible porque las entidades de salud organicen frecuentemente el trabajo con turnos en la mañana y en la noche, lo que permite que muchos puedan trabajar por la mañana en un lugar y al final del día en otro.

**Figura 3:** - Extractos de las entrevistas alusivos a las percepciones de los enfermeros jóvenes sobre las condiciones de empleo (n = 15). Bogotá, Colombia, 2021-2022

*OPS = Orden de prestación de servicios

 Varios participantes manifestaron su deseo de migrar, este código corresponde a la subcategoría de talento humano presentado en la Figura 1 . La principal razón es porque perciben que en países más ricos, como Alemania, Estados Unidos de América o Canadá, además de tener mejores condiciones de vida que Colombia, se ofrecen mejores condiciones laborales y mayor reconocimiento social para sus profesiones. Dentro de los factores que limitan la migración están el desconocimiento de lenguas extranjeras, el arraigo por el país y la familia, la incertidumbre de la migración y el subempleo. 

Para los profesionales en enfermería, las molestias físicas relacionadas con su empleo se pueden asociar con efectos negativos en sus hábitos de vida y prácticas de cuidado diarias. Aparece el estrés como un factor que desencadena dolores de cabeza o de estómago que se asocian con las cargas laborales a las que se ven enfrentados esos profesionales.

La angustia relacionada con la falta de dinero para responder a las obligaciones económicas y la frustración asociada con la ausencia de independencia fueron referidas como unos de los síntomas más frecuentes que afectaron su salud mental.

De igual manera, los entrevistados consideran que el pluriempleo afecta tanto al profesional como a la calidad de los servicios, pues los profesionales duermen menos, están más cansados y con menos disposición al trabajo.

Por lo general, otras molestias físicas como cansancio, dolores musculares y migrañas están presentes en casos de profesionales que tienen dos o más trabajos porque se debe responder a dos jefes, hay un aumento en tiempos de desplazamiento, carga administrativa y mental, poco tiempo para el descanso, el ocio y los hábitos saludables cotidianos.

### Condiciones de trabajo psicosociales

 La Figura 4 muestra los extractos referentes a las condiciones psicosociales identificadas por los participantes, contempladas en la categoría condiciones de trabajo descrita en la Figura 1 . Estas fueron variadas y aludieron a diversos asuntos que representaron fuente de molestia o preocupación. Para empezar, los profesionales señalaron las exigencias mentales de atención, concentración y memoria involucradas en las actividades clínicas y administrativas propias del cuidado de la salud de otras personas como una condición psicosocial relevante. De igual manera, identificaron altas exigencias emocionales producto de la exposición a la enfermedad y muerte, así como por presenciar diariamente el sufrimiento de los pacientes, lo que se intensifica por el vínculo o nexo emocional que se crea con ellos. La alta carga mental y las demandas emocionales fueron asociadas por los participantes con efectos como fatiga, cansancio, frustración y somatización, lo cual se vio especialmente incrementado en pandemia. 

**Figura 4:** - Extractos de las entrevistas alusivos a las percepciones de los enfermeros jóvenes sobre las condiciones psicosociales de trabajo (n = 15). Bogotá, Colombia, 2021-2022

De otro lado, la rotación por turnos de trabajo se asoció a un mayor esfuerzo adaptativo que tiene unas implicaciones negativas en los hábitos y estilo de vida de los profesionales; así como en sus relaciones interpersonales fuera del trabajo. Se identificó la variabilidad en los turnos como un aspecto que dificulta establecer rutinas, mantener buenos hábitos y desarrollar actividades extralaborables.

 Además, se mencionó que las decisiones y cambios administrativos en la organización del trabajo tiene repercusiones negativas para el desarrollo de sus actividades, lo que en muchas ocasiones ha implicado una sobrecarga o una distribución inequitativa del trabajo y esto se asoció a desgaste, fatiga y cansancio, lo que en palabras de los participantes se traduce en sensación de *quemarse* por el trabajo. 

De acuerdo con los profesionales, las relaciones interpersonales con compañeros dentro de la institución donde se labora determinan cómo se desempeñan las actividades, la satisfacción con el trabajo e incluso, su estado de ánimo. Esto debido a que los conflictos entre compañeros y las deficiencias en la comunicación generan un ambiente tenso y estresante. Lo anterior se puede intensificar por factores como la antigüedad y las diferencias de años de experiencia en el trabajo, siendo más difícil la relación con colegas que tienen más antigüedad.

Finalmente, la relación con los jefes inmediatos fue identificada como un aspecto que tiene mucho peso en la satisfacción y bienestar general en el trabajo. Es así como estilos de mando caracterizados por la buena comunicación y el soporte fueron percibidos como muy favorables; en contraposición, deficiencias en la comunicación y la falta de apoyo y respaldo son percibidas como características de los estilos de liderazgo que tienen un impacto negativo entre los profesionales.

### Condiciones de trabajo materiales

 En la Figura 5 se presentan extractos de los participantes correspondientes con las condiciones de trabajo materiales (ver Figura 1 ), en los que se mencionarán los más relevantes. En relación con la exposición al peligro biomecánico, los profesionales manifestaron que su trabajo es frecuentemente en bipedestación seguido de postura sedente en superficies de trabajo inadecuadas, por lo que deben adoptar posturas forzadas y sostenidas durante la jornada con la consecuente presentación de molestias o dolores. 

El esfuerzo y la manipulación de cargas fueron referidas en la movilización de pacientes en situaciones de emergencia o pacientes con limitaciones de movilidad. Además, el movimiento repetitivo fue percibido en dos situaciones: la apertura de las ampollas durante la administración de medicamentos y el uso del computador para las actividades administrativas.

**Figura 5:** - Extractos de las entrevistas y grupo focal alusivos a las percepciones de los enfermeros jóvenes sobre las condiciones materiales de trabajo (n = 15). Bogotá, Colombia, 2021-2022

*KPC = *Klebsiella pneumoniae carbapenemase* ; ^†^ UCI = Unidad de cuidados intensivos

Según la caracterización del peligro biológico, los enfermeros refirieron una normalización de dicha exposición por su quehacer profesional, hubo especial alusión al manejo de pacientes con confirmación de microorganismos multirresistentes como situaciones de riesgo. También mencionaron momentos de incertidumbre, angustia y miedo ante la posibilidad de infectarse cuando se han accidentado durante la manipulación de objetos contaminados.

Los participantes manifestaron que durante la pandemia varios riesgos ocupacionales se agudizaron: exposición altísima al SARS-CoV-2, molestias térmicas debido al hacinamiento de pacientes, insuficientes condiciones de ventilación en las áreas de atención, aumento en la frecuencia del lavado de manos, manipulación de pacientes por su condición crítica que les impide moverse y permanente uso de elementos de protección personal, que causaron cambios en sus hábitos cotidianos.

Otras situaciones riesgosas para la salud de los participantes fueron los productos químicos por lavado de manos, el uso de guantes de látex y el manejo de medicamentos; la exposición a ruido o iluminación inadecuada; el manejo de camas o sillas de ruedas en mal estado y la atención de pacientes e interacción con familiares por posibles agresiones (golpes y mordeduras).

## Discusión

 La caracterización sociodemográfica identificada en el estudio corrobora que en la profesión de enfermería predomina el sexo femenino. Los hallazgos relevantes del estudio relacionado con las percepciones de los enfermeros jóvenes sobre sus condiciones de empleo, trabajo y salud, indicaron que la precarización laboral es característica del sector salud, debido a la baja posibilidad de encontrar un empleo con garantías laborales por el tipo de vinculación y la retribución que se oferta en el mercado laboral ^(^ 5 ^,^ 9 ^)^ , lo que fomenta el pluriempleo en los enfermeros ^(^ 17 ^)^ . 

 A partir del modelo planteado por Benach ^(^ 4 ^)^ que nos permite interpretar las percepciones que tienen los profesionales jóvenes de enfermería de la relación entre condiciones de trabajo, empleo y salud, desde los niveles macro y microestructural, los hallazgos de la investigación permitieron identificar cómo la precariedad del empleo en el sector salud refleja las condiciones del mercado laboral. Llaman la atención dos aspectos que contribuyen en la precarización del trabajo: la contratación por terceros y no directamente por las instituciones de salud, lo cual ha acrecentado la falta de responsabilidad por parte de estas instituciones para garantizar condiciones dignas de empleo, y el aprovechamiento de los empleadores al contratar profesionales jóvenes que tienen la necesidad de acumular experiencia laboral, condiciones que se analizan en otros estudios, donde la edad y ser recién egresado son relevantes en la contratación de servicios en el sector salud ^(^ 3 ^)^ . Lo antes dicho tuvo efectos en la salud de los participantes al mencionar molestias psicológicas como estrés y ansiedad con manifestaciones somáticas como dolores de cabeza y en otras partes del cuerpo e insomnio. 

 Por otra parte, la retribución inferior a sus expectativas como profesionales de enfermería acorde con la carga laboral y el nivel de estudios obtenido ^(^ 18 ^)^ , al igual que el desconocimiento de las condiciones del mercado laboral en los recién egresados, fueron situaciones que les generaron diversas molestias psicológicas como ansiedad o desmotivación. Estos aspectos, los lleva a pensar en migrar con el propósito de mejorar su calidad de vida. 

 Desde la perspectiva de las condiciones de trabajo planteadas por Benach ^(^ 4 ^)^ en el componente microestructural del modelo, las condiciones psicosociales de los enfermeros se caracterizan por la exigencia de desarrollar actividades laborales en turnos rotativos y nocturnos, trabajar en días que comúnmente se destinan al descanso; por laborar durante jornadas diarias prolongadas y extenuantes, y por experimentar frecuentes cambios de horario (extensión del turno de trabajo o modificaciones en los horarios de inicio y finalización de la jornada), lo que crea dificultades para desarrollar actividades personales y extralaborales generando un detrimento en el equilibrio entre la vida familiar, social y laboral ^(^ 3 ^,^ 19 ^-^ 21 ^)^ . 

 La atención directa de pacientes y la interacción frecuente con familiares es una característica distintiva e inherente al ejercicio asistencial de esta profesión, lo que implica una serie de demandas emocionales propias del trabajo con personas en situación de vulnerabilidad. Como elementos particulares de las demandas emocionales se encuentran el cercano enfrentamiento a la enfermedad y la muerte, el manejo de pacientes y familiares cuyos estados emocionales están alterados (nerviosos, temerosos, ansiosos o estresados) por lo que resulta abrumador interactuar con ellos; el requerimiento permanente de comprender y empatizar con las emociones de temor, ansiedad y sufrimiento de pacientes y familiares; y la necesidad de controlar y esconder las propias emociones de miedo, incertidumbre, dolor y frustración que se pueden producir en el desempeño de la ocupación ^(^ 22 ^-^ 23 ^)^ . Cabe anotar que el contacto interpersonal directo con pacientes y familiares propicia un contexto en el que se presentan actos de violencia, expresados de forma muy variada, incluidos el abuso verbal, la intimidación, las amenazas, el acoso sexual y las agresiones físicas ^(^ 24 ^-^ 25 ^)^ . 

 La demanda de carga mental, asociada con las altas exigencias de concentración, atención y memoria que se hacen a los enfermeros en virtud de su trabajo, así como, las exigencias que se derivan del manejo de información detallada y precisa, es otro factor de riesgo psicosocial distintivo, advirtiéndose que cuando se presenta esta condición de forma constante en el trabajo, dicha circunstancia puede asociarse con deficiencias en el desempeño laboral, fatiga o cansancio y mayor probabilidad de cometer errores ^(^ 24 ^)^ . 

 Las relaciones o el contacto que se establece con otras personas en el medio laboral significan para los enfermeros múltiples tipos de interacciones, entre las que se encuentran aquellas que se establecen con jefes o superiores, compañeros de trabajo que se desempeñan en áreas asistenciales o administrativas, pacientes y sus familiares y público externo. Todas ellas pueden significar una fuente de peligro toda vez que suelen desarrollarse bajo presión de tiempo, elevada carga emocional, altos volúmenes de trabajo y horarios laborales atípicos. Este contexto se describe como un ámbito propicio para que se presente un deficiente apoyo social de los jefes y entre compañeros, comunicación ineficaz, dificultades y conflictos en las relaciones interpersonales con jefes, pares o con profesionales de otras áreas de trabajo ^(^ 22 ^-^ 23 ^,^ 25 ^-^ 26 ^)^ . 

 En suma, las condiciones de trabajo materiales en enfermería representan alto riesgo de desarrollar desórdenes musculoesqueléticos (dolores de espalda, cuello, hombros y extremidades inferiores) por la necesidad de levantar y mover a pacientes con limitación en su movilidad, permanecer sentado, caminar y estar de pie durante periodos prolongados, empujar o levantar objetos pesados y equipos, entre otras situaciones ^(^ 27 ^-^ 29 ^)^ . 

 En consonancia con otros estudios, los espacios de trabajo inadecuados o el mal diseño del mobiliario (camillas y sillas) fueron una de las causas de las dolencias, identificando la relación entre lesiones osteomusculares y las condiciones de trabajo ^(^ 29 ^)^ . La presencia de síntomas musculoesqueléticos fue subestimada por los profesionales jóvenes, quienes no los percibieron suficientemente graves o dolorosos como para informar a empleadores, atribuyéndolos a su mala postura y prácticas de trabajo riesgosas ^(^ 30 ^)^ . 

 Otro subtema de interés fue la exposición a los agentes infecciosos por la exposición a sangre y fluidos corporales a pesar de la manifestación dada por los entrevistados de naturalizar en sus actividades cotidianas el contacto permanente y directo con estos microorganismos ^(^ 31 ^)^ . Un estudio fenomenológico realizado con diez enfermeras en Irán mostró el riesgo alto de exposición a enfermedades transmitidas por la sangre y enfermedades nosocomiales ^(^ 28 ^)^ . Los accidentes biológicos por punciones con agujas y objetos cortopunzantes son comunes en los enfermeros ^(^ 32 ^-^ 33 ^)^ , en el presente estudio, todos manifestaron haber tenido al menos un accidente durante su trayectoria laboral ^(^ 33 ^)^ . 

 La percepción de otras condiciones de trabajo como temperatura, iluminación, ruido y manipulación de medicamentos no fueron identificadas tan comúnmente por los participantes a pesar de estar presentes ^(^ 34 ^-^ 35 ^)^ . 

 La pandemia por COVID-19 produjo cambios tanto en las condiciones de empleo como de trabajo en enfermería. Aunque fue notorio un aumento en la oferta laboral para dar respuesta a la atención masiva, los contratos eran por meses, lo que generaba inseguridad económica a largo plazo ^(^ 3 ^)^ . Además, en este periodo los enfermeros mencionaron que la carga laboral se incrementó a pesar de que los salarios y la calidad de empleo se mantuvieron bajos ^(^ 17 ^)^ . 

 Múltiples estudios han demostrado exacerbación de molestias físicas y psicológicas relacionadas con el aumento de la carga laboral y la ampliación de los horarios ^(^ 36 ^-^ 37 ^)^ lo que conllevó cambios en el estilo de vida y de trabajo ^(^ 38 ^-^ 39 ^)^ . Durante la pandemia, las dermatitis o lesiones cutáneas por el lavado de manos intensivo (más de diez veces al día) y el uso de mascarillas durante más de seis horas fueron relevantes ^(^ 32 ^,^ 39 ^)^ y se asociaron con el miedo a infectarse y a contagiar a familiares y amigos ^(^ 35 ^)^ . 

Con respecto a las contribuciones de este estudio en el avance científico para la profesión de enfermería, en primera instancia se reconoce el valor de la investigación cualitativa para la comprensión de la relación trabajo-salud, más allá de la cuantificación del riesgo, rescatando las voces de los enfermeros jóvenes y sus percepciones en las que resaltan preponderantemente los tipos de vinculación y la remuneración, así como los factores psicosociales, siendo los que más afectan su salud. De igual manera, los hallazgos del estudio aportan una mirada integrada entre las condiciones de empleo y las condiciones de trabajo superando la visión tradicional de la seguridad y salud en el trabajo, además de incorporar los factores que reflejan las relaciones de poder entre el gobierno, los empleadores y los profesionales.

A partir de los hallazgos presentados en esta investigación, surge la necesidad de considerar en los procesos de formación de los profesionales de enfermería, la inclusión de espacios en el currículo orientados a comprender los factores que determinan la inserción laboral de los recién egresados, con el fin de prepararlos para enfrentarse al mercado laboral. Asimismo, se advierte la necesidad de entrenarlos para llevar a cabo acciones de prevención frente a la exposición a condiciones psicosociales, biomecánicas, biológicas y de seguridad en sus lugares de trabajo, siendo estas prioritarias en el sector salud.

Cabe destacar que el análisis por duplas y la conformación de un equipo interdisciplinario cuya experiencia en el ámbito asistencial es reducida, permitió poner en primer plano el discurso de los participantes y facilitó las reflexiones sobre sus condiciones laborales y el ser joven en el mercado laboral desde diversas perspectivas.

El alcance y planteamiento metodológico del estudio representó algunas limitaciones que pueden ser superadas en investigaciones posteriores, entre ellas: a) en la muestra no se contó con mayor diversidad respecto a los tipos de contratación para enriquecer los hallazgos; b) la falta de representatividad de enfermeros en otras regiones de Colombia, siendo que las condiciones de empleo y trabajo pueden variar sustancialmente; c) transversalidad de la reflexivilidad personal en el equipo investigador para declarar la influencia en la recopilación y análisis de datos más allá de la perspectiva profesional; d) la realización remota del grupo focal; e) el reconocimiento de los hallazgos de los participantes de manera individual.

## Conclusión

La mayoría de los participantes consideraron que su trabajo ha afectado negativamente su salud mental y emocional, su salud física, sus hábitos de vida saludable, lo que genera estrés o ansiedad, sentimientos de incertidumbre e inseguridad laboral por la inestabilidad de las contrataciones.

Los trabajadores jóvenes suelen aceptar las oportunidades laborales por la necesidad de certificar experiencia laboral. Se menciona la importancia que tiene la estabilidad laboral a la hora de elegir un empleo, pues, además de brindar tranquilidad, posibilita su independencia con mayor rapidez.

Con respecto a las condiciones de trabajo, las psicosociales fueron las más relevantes para los profesionales en la medida que las jornadas de trabajo, los turnos rotativos y nocturnos al igual que la interacción con otras personas y las demandas de carga mental alteran el desarrollo de actividades laborales, personales y extralaborales, las cuales se exacerbaron en la pandemia por COVID-19.

# Translation: Percepções de jovens enfermeiros sobre sua condição de emprego, de trabalho e de saúde *

**DOI:** 10.1590/1518-8345.7398.4336

**Published:** 2024-09-23

**Authors:** Mabel Rocío Hernández Díaz, Zuly Bibiana Suárez Morales, Angelica María Vargas Monroy, Andrey Sebastián Castiblanco Prieto

**Affiliations:** ^1^ Pontificia Universidad Javeriana, Instituto de Salud Pública, Bogotá, DC, Colombia

**Keywords:** Enfermagem, Condições de trabalho, Saúde Ocupacional, Emprego, Setor de Saúde, Colômbia

## Abstract

**Objetivo:**

interpretar as percepções que os jovens profissionais de enfermagem têm sobre a relação entre condições de trabalho, emprego e saúde.

**Método:**

estudo qualitativo com abordagem interpretativa das experiências de trabalho de quinze jovens enfermeiros, que participaram da pesquisa por meio de amostragem voluntária em bola de neve. Realizou-se a análise dos dados das entrevistas e grupo focal para alcançar uma aproximação à realidade da vida profissional.

**Resultados:**

os achados relevantes do estudo indicaram que a precarização no trabalho é característica desse grupo populacional, principalmente no que diz respeito ao tipo de contratação e à remuneração recebida. As condições psicossociais de trabalho foram as que mais provocaram efeitos na saúde mental e física segundo os participantes, sendo essas condições agravadas durante a pandemia da COVID-19.

**Conclusão:**

este estudo apresenta, na perspectiva de jovens enfermeiros, como são percebidas as características macro e microestruturais do trabalho e sua relação com a saúde, apontando os elementos-chave para gerar intervenções com abordagem de curso de vida que divulguem os postulados do trabalho decente e ambiente saudável em seus ambientes de trabalho, bem como ações para prevenir lesões ou danos à saúde dos enfermeiros.

## Destaques

**(1)** Os jovens enfermeiros percebem uma significativa precarização do trabalho.

**(2)** As modalidades de contratação e remuneração são percebidas como as condições mais desfavoráveis.

**(3)** Os participantes indicam que o emprego e as condições de trabalho afetam a sua saúde.

**(4)** As condições psicossociais de trabalho são as mais prejudiciais segundo os participantes.

## Introdução

 Nas últimas décadas, houve um agravamento da situação do mercado de trabalho na América Latina e no Caribe que afeta os jovens. A má qualidade dos empregos para este grupo populacional manifesta-se em condições de trabalho precárias ^(^ 1 ^)^ , na falta de proteção legal e social e em oportunidades limitadas de formação e desenvolvimento profissional ^(^ 2 ^-^ 3 ^)^ . 

 A referência conceitual deste estudo baseia-se no modelo proposto por Benach ^(^ 4 ^)^ da relação trabalho-saúde nos níveis macro e microestrutural. No nível macro, as relações de poder entre governos, empresas, grupos representativos dos trabalhadores e da sociedade desempenham um papel central na geração de políticas que regulam o mercado de trabalho e as condições de bem-estar dos trabalhadores, moldando as condições de emprego; ao passo que o nível micro, ou seja, as condições de trabalho, explica as exposições diferenciais de natureza psicossocial ou material e sua relação com a saúde. 

 No que se refere a algumas condições de trabalho e emprego que afetam particularmente os jovens trabalhadores do sector da saúde, encontramos: empregos instáveis devido à terceirização e contratos temporários, sendo que muitas vezes não são devidamente supervisionados ^(^ 5 ^)^ ; decisões equivocadas por eles tomadas por falta de representação e desconhecimento de seus direitos trabalhistas, o que pode provocar a tendência a aceitar condições de trabalho desfavoráveis ^(^ 2 ^)^ ; e a falta de oportunidades de qualificação e formação para o trabalho, o que indica que um jovem ao ingressar pela primeira vez no mercado de trabalho não possui as mesmas competências e experiência que um trabalhador adulto, razão pela qual ele está mais inclinado a aceitar condições adversas de trabalho e emprego ^(^ 3 ^)^ que configuram maior flexibilidade laboral e terceirização da relação de trabalho ^(^ 6 ^-^ 7 ^)^ . 

O exposto poderá tornar-se um precedente importante que marcará, num futuro próximo, o déficit de talento humano no setor e o detrimento da qualidade da prestação de serviços, o que implica um potencial nocivo para os sistemas de saúde.

 Diversas investigações realizadas sobre precárias condições de trabalho e emprego em profissionais de saúde indicam que sua saúde, segurança e qualidade de vida são cada vez mais afetadas ^(^ 8 ^-^ 11 ^)^ , porém, estas carecem da perspectiva dos jovens trabalhadores da área de cuidados à saúde humana formados recentemente. Surge então a necessidade de interpretar as percepções que os jovens profissionais de enfermagem têm da relação entre condições de trabalho, emprego e saúde, e desta forma tornar visíveis as condições de trabalho que mais os afetam, para fornecer informações aos diferentes atores do sistema de saúde, que permitam gerar ações visando melhorar essas condições. 

## Método

### Tipo de estudo

 Pesquisa qualitativa, com abordagem interpretativa na perspectiva de Weber ^(^ 12 ^)^ , que abarca alguns princípios fenomenológicos com base nas experiências de vida na perspectiva do sujeito, no que diz respeito ao seu vínculo empregatício, às condições de emprego e trabalho vivenciadas e como estas afetam a saúde. O manuscrito seguiu as recomendações do *checklist* de critérios COREQ ( *Consolidated Criteria for Reporting Qualitative Research* ) ^(^ 13 ^)^ . 

### Local e período de coleta de dados

O estudo foi realizado em Bogotá, Colômbia, de agosto de 2021 a fevereiro de 2022.

### Participantes

Com o objetivo de buscar a participação voluntária de homens e mulheres, que se dispusessem a compartilhar suas experiências, levando em consideração a condição de ser jovem profissional, foi realizada uma convocatória aberta aos profissionais de enfermagem por meio de amostragem voluntária e pelo método bola de neve, por meio de divulgação em agremiações, faculdades de enfermagem e redes de egressos. Foi possível contar com a participação de 15 enfermeiros, que não tinham vínculo prévio com os pesquisadores e atenderam aos seguintes critérios: ser profissional graduado em enfermagem, ter entre 21 e 28 anos de idade, ter trabalhado em Bogotá, D.C, com mínimo de 4 meses e máximo de 5 anos de experiência profissional.

### Instrumentos para coleta de dados

 Foram utilizados dois instrumentos para coleta de dados submetidos a um teste piloto. Este teste foi aplicado a três entrevistados e a um grupo focal no projeto marco desta pesquisa, que incluiu cinco profissões de saúde diferentes da enfermagem. Os dados resultantes do teste-piloto foram utilizados para ajuste metodológico do estudo. A construção das questões necessárias para a coleta de dados resultou da revisão da literatura e do modelo Benach ^(^ 4 ^)^ que emergiu de um exercício analítico e reflexivo dos investigadores. 

Entrevistas semiestruturadas: foi elaborado um guia de questões centrais sobre seu histórico de trabalho como enfermeiro assistencial, notadamente, sobre as características do trabalho, sua rotina de trabalho e os efeitos em sua saúde, dando espaço ao entrevistador para orientar a conversa, aprofundando os elementos que gerassem mais reações nos participantes, conforme necessário. As perguntas realizadas evitavam induzir respostas, eram neutras e permitiam que os participantes narrassem suas experiências e pontos de vista sem emitir críticas ou julgamentos a esse respeito. Cada entrevista durou em média 120 minutos;

 Grupo focal: havia um guia de orientação ^(^ 14 ^)^ para aprofundar e complementar aspectos sobre as condições de emprego, trabalho e saúde que os participantes enfrentaram na vida profissional, criando um espaço de conversa entre os participantes. A duração do grupo focal foi de 180 minutos. 

### Coleta de dados

À medida que ocorria o processo de divulgação da convocatória, a amostra voluntária foi formada por conveniência, sendo este o primeiro momento de coleta. Foram realizadas 10 entrevistas individuais, agendadas fora do horário de trabalho, criando um ambiente tranquilo e sem interrupções, o qual facilitou o diálogo e a troca de informações com confiança e empatia. A partir dos elementos centrais identificados nas entrevistas, foi realizado um grupo focal com a participação de seis enfermeiros, para aprofundar e complementar as informações coletadas. Vale evidenciar que um dos participantes do grupo focal já havia sido entrevistado anteriormente. As entrevistas e o grupo focal foram conduzidos por dois pesquisadores, um responsável pela condução e outro pelo registro. Em todos os casos, foi garantido que um dos membros tivesse formação em saúde e segurança no trabalho e o outro em ciências sociais para promover a integração de diferentes perspectivas disciplinares.

 Todos os profissionais assinaram previamente um termo de consentimento livre e esclarecido, no qual manifestaram livre e voluntariamente o interesse em participar do estudo. Por sua parte, a equipe de pesquisa garantiu as condições de anonimato, confidencialidade e utilização científica dos dados coletados. Os instrumentos de coleta de dados foram implementados seguindo as diretrizes colombianas de proteção de dados, indicando-se no consentimento informado a política da *Universidad Javeriana* para a proteção de dados pessoais em cumprimento à Lei 1.581 de 2012 ( *Habeas Data* ). 

 Devido ao contexto situacional da crise sanitária da COVID-19, todas as entrevistas e o grupo focal foram realizados remotamente por meio da ferramenta *Microsoft Teams* ^®^ , o que permitiu sua gravação com acesso apenas para o pesquisador principal. A transcrição literal das entrevistas e do grupo focal utilizou o *plugin* *NVivo* *Transcription* ^®^ , posteriormente foi realizada uma revisão desses documentos por duplas de pesquisadores. 

A coleta de dados revalidou-se de maneira remota, atendendo cuidadosamente às condições pandémicas. Os autores formularam reflexões sobre as implicações que essas investigações poderiam ter sobre os sujeitos em relação a questões como: 1. a garantia na utilização de seus dados; 2. a aplicação dos resultados obtidos e 3. o respeito pela privacidade de suas experiências compartilhadas. Abordar essas questões continuará sendo uma tarefa permanente na pesquisa qualitativa.

### Processamento e análise de dados

 A análise de conteúdo ^(^ 15 ^)^ baseada na redução e reelaboração dos dados surgiu da formulação de códigos que formaram conjuntos de agrupamentos homogêneos. Os códigos foram integrados em categorias para estabelecer relações entre eles, utilizando codificação axial e seletiva ^(^ 16 ^)^ . 

 A codificação dos conteúdos das entrevistas e do grupo focal foi realizada pelos membros da equipe de pesquisa em duplas, utilizando *o NVivo 11* ^®^ para chegar a um consenso e assim validar o resultado desta atividade. Em seguida, foram identificadas relações por concorrência dos códigos para identificar semelhanças e diferenças nas percepções e assim estabelecer a saturação das informações. 

 A Figura 1 apresenta as categorias e subcategorias adaptadas do modelo de Benach ^(^ 4 ^)^ para a codificação dos dados coletados. Neste modelo, são definidas as condições de emprego, bem como as circunstâncias em que uma pessoa exerce um trabalho ou ocupação. Frequentemente pressupõe a existência de um acordo ou relação entre um empregador que contrata trabalhadores e um empregado que oferece a sua força de trabalho e as condições de trabalho que contemplam os elementos, agentes ou fatores inerentes à atividade laboral que influenciam significativamente a geração de riscos para a segurança e a saúde dos trabalhadores. 

**Figura 1:** - Categorias e subcategorias do estudo

Neste estudo foi identificada a categoria emergente denominada pandemia, na qual foram consolidadas as características relatadas pelos entrevistados relacionadas à crise sanitária da COVID-19.

Após a codificação das informações, os dados obtidos em diferentes momentos e espaços foram interpretados junto à equipe interdisciplinar de pesquisa, atingindo-se a saturação quando alcançadas a densidade e autenticidade das informações. Os dados coletados foram triangulados, obtendo-se informações descritivas a partir das narrativas dos participantes, que foram analisadas à luz da literatura pertinente em torno da relação entre emprego, trabalho e condições de saúde. Os participantes foram convidados para um evento de socialização em que foram divulgados os resultados consolidados das entrevistas, abrindo espaço para discussão em pequenos grupos, realizando-se uma jornada exclusiva para os profissionais de enfermagem pela Organização Colegiada de Enfermagem da Colômbia.

### Aspectos éticos

A pesquisa foi enquadrada nas considerações éticas estabelecidas pela Declaração de Helsinque (1964) e pela Resolução 8.430 de 1993 do Ministério da Saúde e Proteção Social da Colômbia, em relação à participação voluntária, à confidencialidade das informações coletadas, à aplicação de consentimento informado e armazenamento das informações obtidas.

O trabalho de campo foi desenvolvido considerando os princípios de segurança, consentimento voluntário e autonomia. Os dados coletados foram tratados de forma confidencial, anônima e apenas para fins de pesquisa, eliminando-se os arquivos uma vez transcritos.

 O projeto de pesquisa foi aprovado pelo Comitê de Ética e Pesquisa do *Instituto de Salud Pública de la Pontifícia Universidad Javeriana* – Bogotá, Colômbia. 

## Resultados

### Características sociodemográficas e ocupacionais

 A Figura 2 apresenta as características da pesquisa da qual participaram 15 profissionais de enfermagem, 73% deles identificados como do sexo feminino e solteiros, a idade média foi de 25,8 anos (± 2) e 47% tinham experiência profissional superior a quatro anos. 

**Figura 2:** - Características sociodemográficas dos jovens enfermeiros participantes (n = 15). Bogotá, Colômbia, 2021-2022

 Do ponto de vista ocupacional, constatou-se que a renda mensal variou entre US$ 452 a 904 ^1^ , a jornada de trabalho tinha uma variação diária de 6 a 12 horas e as áreas de trabalho mais frequentes foram internação, emergências, unidades de terapia intensiva e consulta ambulatorial. 

### Condições de contratação

 No que diz respeito à subcategoria qualidade do emprego descrita na Figura 1 , a maioria dos entrevistados comentou que nos seus locais de trabalho mais de 80% dos jovens profissionais estão vinculados a contratos de prestação de serviços ^2^ , apesar de nenhum deles ter este tipo de contrato (ver as referências relacionadas na Figura 3 ). Expressam também a ausência de benefícios sociais como férias remuneradas, gratificações, verbas rescisórias e outros benefícios que limitam seus projetos futuros e seu potencial de poupança. 

No grupo focal, constatou-se a percepção geral de que estes são os tipos de contratos com mais desvantagens e mais comuns. Entre as desvantagens são mencionadas a instabilidade laboral, a falta de férias remuneradas e a impossibilidade de poupar e de obter crédito.

 Dentro dos códigos relativos à subcategoria mercado de trabalho conforme a Figura 1 , o acesso ao emprego e à remuneração foram os mais citados. Para os entrevistados, ser jovem determina as suas possibilidades de acesso a um emprego justo. Eles percebem que os empregos bem remunerados exigem uma experiência que, devido à idade, torna-se impossível de obter. Isso fecha as portas, inclusive, no que se refere a vagas para as quais se consideram qualificados, levando-os a aceitar ofertas de empregos precários. 

Em geral, os profissionais entrevistados consideram que a remuneração financeira que recebem pelo seu trabalho é baixa levando em consideração o número de atividades que desempenham e a responsabilidade por elas. Mesmo aqueles que se sentem satisfeitos com sua renda costumam dizer que, quando comparados com o que ganham seus conhecidos, as profissões da saúde são mal remuneradas.

 A pluralidade de trabalho foi a situação mais discutida entre os participantes, indicando que esta é uma atividade comum entre os profissionais do setor da saúde. Quase todas as pessoas entrevistadas tiveram essa experiência ou conheciam alguém que a teve. Aliás, um dos entrevistados afirmou que esta atividade é coloquialmente conhecida como *turnear* . 

Os profissionais que assumem dois empregos fazem isso pois a remuneração dos empregos costuma ser baixa e há instabilidade laboral. Além do mais, isso é possível porque as entidades de saúde organizam frequentemente o trabalho com turnos matinais e noturnos, o que permite que muitos trabalhem de manhã num local e ao final do dia em outro.

**Figura 3:** - Trechos das entrevistas referentes às percepções dos jovens enfermeiros sobre as condições de emprego (n = 15). Bogotá, Colômbia, 2021-2022

*OPS = Ordem de Prestação de Serviço

 Vários participantes manifestaram o desejo de migrar; este código corresponde à subcategoria de talento humano apresentada na Figura 1 . A principal razão é porque percebem que países mais ricos, como a Alemanha, os Estados Unidos da América ou o Canadá, além de terem uma vida melhor do que na Colômbia, oferecem melhores condições de trabalho e maior reconhecimento social para suas profissões. Entre os fatores que limitam a migração estão a falta de conhecimento de línguas estrangeiras, as raízes no país e na família, a incerteza da migração e o subemprego. 

Para os profissionais de enfermagem, o desconforto físico relacionado ao seu emprego pode estar associado a efeitos negativos nos seus hábitos de vida diária e práticas de cuidado. O estresse aparece como um fator desencadeante de dores de cabeça ou de estômago que estão associadas à carga de trabalho que esses profissionais enfrentam.

A angústia relacionada com a falta de dinheiro para afrontar obrigações financeiras e a frustração associada à falta de independência foram referidas como alguns dos sintomas mais frequentes que afetaram a sua saúde mental.

Da mesma forma, os entrevistados consideram que o a pluralidade de trabalho afeta tanto o profissional quanto a qualidade dos serviços, uma vez que os profissionais dormem menos, estão mais cansados e menos dispostos a trabalhar.

Geralmente, outros desconfortos físicos como cansaço, dores musculares e enxaquecas estão presentes em casos de profissionais que possuem dois ou mais empregos porque devem responder a dois chefes, há aumento do tempo de deslocamento, carga administrativa e mental, pouco tempo para descanso, lazer e hábitos saudáveis cotidianos.

### Condições psicossociais de trabalho

 A Figura 4 apresenta os extratos referentes às condições psicossociais identificadas pelos participantes, contempladas na categoria de condições de trabalho descrita na Figura 1 . Estas eram variadas e referiam-se a questões diversas que representavam fonte de desconforto ou preocupação. Inicialmente, os profissionais apontaram as demandas mentais de atenção, concentração e memória envolvidas nas atividades clínicas e administrativas de cuidado da saúde alheia como condição psicossocial relevante. Da mesma forma, identificaram elevadas demandas emocionais em decorrência da exposição ao adoecimento e à morte, bem como pelo fato de presenciar diariamente o sofrimento dos pacientes, que é intensificado pelo vínculo ou conexão afetiva que se cria com eles. A alta carga mental e as demandas emocionais foram associadas pelos participantes a efeitos como fadiga, cansaço, frustração e somatização, que aumentaram especialmente durante a pandemia. 

**Figura 4:** - Trechos das entrevistas referentes às percepções dos jovens enfermeiros sobre as condições psicossociais de trabalho (n = 15). Bogotá, Colômbia, 2021-2022

Por outro lado, a rotação do trabalho por turnos esteve associada a um maior esforço adaptativo que tem implicações negativas nos hábitos e estilo de vida dos profissionais, bem como em suas relações interpessoais fora do trabalho. A variabilidade nos turnos foi identificada como um aspecto que dificulta o estabelecimento de rotinas, a manutenção de bons hábitos e o desenvolvimento de atividades extralaborais.

Além disso, foi mencionado que as decisões administrativas e as mudanças na organização do trabalho têm repercussões negativas no desenvolvimento das suas atividades, o que em muitas ocasiões tem implicado uma sobrecarga ou uma distribuição desigual do trabalho e isso está associado ao esgotamento, à fadiga e ao cansaço, o que nas palavras dos participantes se traduz numa sensação de esgotamento pelo trabalho.

Segundo os profissionais, as relações interpessoais com os colegas da instituição onde você trabalha determinam a forma como as atividades são realizadas, a satisfação com o trabalho e até mesmo o seu estado de espírito. Isso porque os conflitos entre colegas e as deficiências na comunicação geram um ambiente tenso e estressante. O acima exposto pode ser intensificado por fatores como maior tempo de trabalho e as diferenças no que diz respeito aos anos de experiência no trabalho, dificultando o relacionamento com os colegas com maior tempo de trabalho.

Por fim, o relacionamento com os chefes imediatos foi identificado como um aspecto que tem muito peso na satisfação geral e no bem-estar no trabalho. Foi assim que os estilos de comando caracterizados por boa comunicação e apoio foram considerados muito favoráveis; em contrapartida, as deficiências de comunicação e a falta de apoio e suporte são percebidas como características de estilos de liderança que impactam negativamente entre os profissionais.

### Condições materiais de trabalho

 A Figura 5 apresenta trechos das entrevistas dos participantes correspondentes às condições materiais de trabalho (ver Figura 1 ), nos quais serão citadas as mais relevantes. Em relação à exposição ao perigo biomecânico, os profissionais afirmaram que seu trabalho é frequentemente realizado em pé seguido de posição sentada em superfícies de trabalho inadequadas, razão pela qual devem adotar posturas forçadas e sustentadas durante o dia com o consequente surgimento de desconforto ou dor. 

O esforço e a manipulação de cargas foram referidos na mobilização de pacientes em situações de emergência ou pacientes com limitações de mobilidade. Além disso, foram percebidos movimentos repetitivos em duas situações: na abertura das ampolas durante a administração de medicamentos e no uso do computador para atividades administrativas.

**Figura 5:** - Trechos das entrevistas e grupo focal alusivos às percepções dos jovens enfermeiros sobre as condições materiais de trabalho (n = 15). Bogotá, Colômbia, 2021-2022

*KPC = *Klebsiella pneumoniae carbapenemase* ; ^†^ UTI = Unidade de Terapia Intensiva

De acordo com a caracterização do risco biológico, os enfermeiros relataram uma normalização da referida exposição devido às suas atribuições profissionais, houve especial referência ao manejo de pacientes com confirmação de microrganismos multirresistentes como situações de risco. Os enfermeiros mencionaram também momentos de incerteza, angústia e medo pela possibilidade de se infectarem ao sofrerem um acidente ao manipular objetos contaminados.

Os participantes afirmaram que durante a pandemia vários riscos ocupacionais tornaram-se mais agudos: exposição muito elevada ao SARS-CoV-2, desconforto térmico devido à sobrelotação de pacientes, condições de ventilação insuficientes nas áreas de cuidados, aumento da frequência de lavagem das mãos, manipulação de pacientes devido a seu estado crítico que os impede de se movimentar e o uso permanente de elementos de proteção individual, o que ocasionou alterações em seus hábitos diários.

Outras situações de risco à saúde dos participantes foram o uso de produtos químicos na lavagem das mãos, o uso de luvas de látex e manuseio de medicamentos; a exposição a ruído ou iluminação inadequada; o manejo de leitos ou cadeiras de rodas em mau estado e o atendimento aos pacientes e interação com familiares devido a possíveis agressões (pancadas e mordidas).

## Discussão

 A caracterização sociodemográfica identificada no estudo corrobora que o sexo feminino predomina na profissão de enfermagem. Os achados relevantes do estudo, relacionados às percepções dos jovens enfermeiros sobre suas condições desemprego, de trabalho e de saúde, indicaram que a precarização no trabalho é característica do setor saúde, devido à baixa possibilidade de encontrar um emprego com garantias laborais por conta do tipo de contratação e da remuneração oferecida no mercado de trabalho ^(^ 5 ^,^ 9 ^)^ , o que incentiva a pluralidade de empregos entre os enfermeiros ^(^ 17 ^)^ . 

 Com base no modelo proposto por Benach ^(^ 4 ^)^ que permite interpretar as percepções que os jovens profissionais de enfermagem têm da relação entre condições de trabalho, emprego e saúde, a partir dos níveis macro e microestrutural, os resultados da pesquisa permitiram identificar o modo como a precariedade do emprego no setor da saúde reflete as condições do mercado de trabalho. Chamam a atenção dois aspectos que contribuem para a precarização do trabalho: a contratação por terceiros e não diretamente pelas instituições de saúde, o que tem aumentado a falta de responsabilidade dessas instituições em garantir condições de trabalho dignas, e o aproveitamento dos empregadores na contratação de jovens profissionais que precisam acumular experiência profissional, condições analisadas em outros estudos, onde a idade e o fato de ser recém-formado são relevantes na contratação de serviços no setor saúde ^(^ 3 ^)^ . O exposto repercutiu na saúde dos participantes ao mencionarem desconfortos psicológicos como estresse e ansiedade com manifestações somáticas como dores de cabeça e em outras partes do corpo e insônia. 

 Por outro lado, a remuneração inferior às suas expectativas como profissionais de enfermagem de acordo com a carga horária e o nível de estudos obtidos ^(^ 18 ^)^ , bem como o desconhecimento das condições do mercado de trabalho nos recém-formados, foram situações que geraram diversos desconfortos psicológicos, como ansiedade ou desmotivação. Esses aspectos os levam a pensar em migrar com o objetivo de melhorar sua qualidade de vida. 

 Na perspectiva das condições de trabalho propostas por Benach ^(^ 4 ^)^ , no componente microestrutural do modelo, as condições psicossociais dos enfermeiros são caracterizadas pela exigência de realizar atividades laborais em turnos rotativos e noturnos, trabalhar em dias comumente utilizados para descanso; trabalhar em jornadas diárias longas e exaustivas e por vivenciarem frequentes mudanças de horário (ampliação do turno de trabalho ou modificações nos horários de início e término do dia), o que dificulta o desenvolvimento de atividades pessoais e extralaborais, ocasionando prejuízo no equilíbrio entre vida familiar, social e profissional ^(^ 3 ^,^ 19 ^-^ 21 ^)^ . 

 O atendimento direto ao paciente e a interação frequente com os familiares é uma característica distintiva e inerente à prática assistencial dessa profissão, que implica uma série de demandas emocionais típicas do trabalho com pessoas em situação de vulnerabilidade. Elementos particulares das exigências emocionais incluem o confronto próximo com a doença e a morte, a gestão de pacientes e familiares cujos estados emocionais estão alterados (nervosos, temerosos, ansiosos ou estressados), tornando-se difícil interagir com eles; a exigência permanente de compreensão e empatia com as emoções de medo, ansiedade e sofrimento dos pacientes e familiares e a necessidade de controlar e esconder as próprias emoções de medo, incerteza, dor e frustração que podem ocorrer durante o desempenho do trabalho ^(^ 22 ^-^ 23 ^)^ . Destaca-se que o contato interpessoal direto com pacientes e familiares fomenta um contexto em que ocorrem atos de violência, expressos de forma muito variada, incluindo abuso verbal, intimidação, ameaças, assédio sexual e violência física ^(^ 24 ^-^ 25 ^)^ . 

 A carga mental, associada à elevada exigência de concentração, atenção e memória imposta aos enfermeiros em virtude do seu trabalho, bem como, as exigências que surgem da gestão de informação detalhada e específica, é outro fator de risco psicossocial distintivo, alertando que quando esta condição ocorre de forma constante no trabalho, esta circunstância pode estar associada a deficiências no desempenho laboral, fadiga ou cansaço e maior probabilidade de cometer erros ^(^ 24 ^)^ . 

 As relações ou contatos estabelecidos com outras pessoas no ambiente de trabalho significam para o enfermeiro múltiplos tipos de interações, entre as quais estão aquelas estabelecidas com chefes ou superiores, colegas de trabalho que atuam na área assistencial ou administrativa, pacientes e seus familiares e o público externo. Todos eles podem ser uma fonte de perigo, pois tendem a desenvolver-se sob pressão de tempo, elevada carga emocional, grandes volumes de trabalho e horários de trabalho atípicos. Esse contexto é descrito como um ambiente propício para o apoio social deficiente da parte dos chefes e entre colegas, comunicação ineficaz, dificuldades e conflitos nas relações interpessoais com seus superiores, seus pares ou com profissionais de outras áreas de trabalho ^(^ 22 ^-^ 23 ^,^ 25 ^-^ 26 ^)^ . 

 Em resumo, as condições materiais de trabalho na enfermagem representam alto risco de desenvolvimento de distúrbios musculoesqueléticos (dores nas costas, pescoço, ombros e membros inferiores) devido à necessidade de levantar e movimentar pacientes com dificuldade de locomoção, permanecer sentado, caminhar e ficar em pé por períodos prolongados, empurrar ou levantar objetos e equipamentos pesados, entre outras situações ^(^ 27 ^-^ 29 ^)^ . 

 Em consonância com outros estudos, espaços de trabalho inadequados ou *design* inadequado de mobiliário (macas e cadeiras) foram uma das causas dos agravos, identificando a relação entre lesões musculoesqueléticas e condições de trabalho ^(^ 29 ^)^ . A presença de sintomas osteomusculares foi subestimada pelos jovens profissionais, que não os percebiam como graves ou suficientemente dolorosos para serem comunicados aos empregadores, atribuindo-os à sua má postura e práticas de trabalho arriscadas ^(^ 30 ^)^ . 

 Outro subtema de interesse foi a exposição a agentes infecciosos como o sangue e os fluidos corporais, apesar da afirmação dos entrevistados de naturalizarem o contato permanente e direto com esses microrganismos em suas atividades diárias ^(^ 31 ^)^ . Um estudo fenomenológico realizado com dez enfermeiros no Irã mostrou o alto risco de exposição a doenças transmitidas pelo sangue e doenças nosocomiais ^(^ 28 ^)^ . Acidentes biológicos por perfurações com agulhas e objetos perfurocortantes são comuns em enfermeiros ^(^ 32 ^-^ 33 ^)^ ; no presente estudo, todos afirmaram ter sofrido pelo menos um acidente durante a carreira profissional ^(^ 33 ^)^ . 

 A percepção de outras condições de trabalho como temperatura, iluminação, ruído e manuseio de medicamentos não foram identificadas habitualmente pelos participantes, apesar de estarem presentes ^(^ 34 ^-^ 35 ^)^ . 

 A pandemia de Covid-19 produziu alterações tanto no emprego como nas condições de trabalho na enfermagem. Embora tenha havido um aumento notável na oferta de trabalho para responder à atenção massiva, os contratos duraram meses, o que gerou insegurança econômica a longo prazo ^(^ 3 ^)^ . Além disso, nesse período, os enfermeiros mencionaram que a carga de trabalho aumentou, embora os salários e a qualidade do emprego permanecessem baixos ^(^ 17 ^)^ . 

 Vários estudos demonstraram exacerbação do desconforto físico e psicológico relacionado ao aumento da carga de trabalho e jornadas mais longas ^(^ 36 ^-^ 37 ^)^ , o que levou a mudanças no estilo de vida e no trabalho ^(^ 38 ^-^ 39 ^)^ . Durante a pandemia, as dermatites ou lesões cutâneas decorrentes da lavagem intensiva das mãos (mais de dez vezes ao dia) e do uso de máscaras por mais de seis horas foram relevantes ^(^ 32 ^,^ 39 ^)^ sendo associadas ao medo de infecção e contágio a familiares e amigos ^(^ 35 ^)^ . 

No que diz respeito às contribuições deste estudo no avanço científico para a profissão de enfermagem, reconhece-se, em primeiro lugar, o valor da pesquisa qualitativa para a compreensão da relação trabalho-saúde, para além da quantificação do risco, resgatando as vozes dos jovens enfermeiros e suas percepções em que se destacam predominantemente os tipos de vínculo empregatício e a remuneração, bem como os fatores psicossociais, sendo os que mais afetam a sua saúde. Da mesma forma, os resultados do estudo proporcionam uma visão integrada entre condições de emprego e condições de trabalho, indo além da visão tradicional de segurança e saúde no trabalho, além de incorporar fatores que refletem as relações de poder entre o governo, os empregadores e os profissionais.

A partir dos achados apresentados nesta pesquisa, surge a necessidade de considerar nos processos de formação dos profissionais de enfermagem, a inclusão de espaços no currículo voltados à compreensão dos fatores que determinam a inserção laboral dos recém-formados, com o objetivo de prepará-los para enfrentar o mercado de trabalho. Da mesma forma, há necessidade de capacitá-los para a realização de ações preventivas contra a exposição a condições psicossociais, biomecânicas, biológicas e de segurança nos seus locais de trabalho, sendo estas prioridades no setor da saúde.

Ressalta-se que a análise por duplas e a formação de uma equipe interdisciplinar com experiência limitada na área da saúde permitiram trazer à tona o discurso dos participantes e facilitaram reflexões sobre suas condições de trabalho e o ser jovem no mercado de trabalho a partir de diversas perspectivas.

A abrangência e a abordagem metodológica do estudo representaram algumas limitações que podem ser superadas em pesquisas posteriores, entre elas: a) a amostra não apresentou maior diversidade quanto aos tipos de contratação para enriquecer os achados; b) a falta de representatividade dos enfermeiros em outras regiões da Colômbia, uma vez que o emprego e as condições de trabalho podem variar substancialmente; c) transversalidade da reflexividade pessoal na equipe de pesquisa para declarar a influência na coleta e análise de dados para além da perspectiva profissional; d) a condução remota do grupo focal e e) o reconhecimento das conclusões dos participantes de forma individual.

## Conclusão

A maioria dos participantes considerou que o seu trabalho tem afetado negativamente a sua saúde mental e emocional, a sua saúde física, os seus hábitos de vida saudáveis, o que gera estresse ou ansiedade, sentimentos de incerteza e insegurança laboral devido à instabilidade das contratações.

Os jovens trabalhadores geralmente aceitam oportunidades de emprego devido à necessidade de certificar experiência profissional. Destaca-se a importância da estabilidade no emprego na hora de escolher um emprego, pois, além de proporcionar tranquilidade, possibilita sua independência com mais rapidez.

No que diz respeito às condições de trabalho, as condições psicossociais foram as mais relevantes para os profissionais na medida em que a jornada de trabalho, os turnos rotativos e noturnos, bem como a interação com outras pessoas e as demandas de carga mental alteram o desenvolvimento das atividades laborais, pessoais e extralaborais, que foram agravadas pela pandemia de COVID-19.
